# Research on the Impact Toughness of 3D-Printed CoCrMo Alloy Components Based on Fractal Theory

**DOI:** 10.3390/biomimetics10050292

**Published:** 2025-05-06

**Authors:** Guoqing Zhang, Junxin Li, Han Wang, Congcong Shangguan, Juanjuan Xie, Yongsheng Zhou

**Affiliations:** 1 School of Mechanical and Electrical Engineering, Zhoukou Normal University, Zhoukou 466000, China; lijunxin1995@163.com (J.L.); 16616zjq@gmail.com (H.W.); xiejuan119@163.com (J.X.); series6685@gmail.com (Y.Z.); 2 Veterinary Laboratory, Shangzhou District Animal Health Supervision Institute, Shangluo 726000, China; szqnyj@163.com

**Keywords:** 3D printing, mechanical properties, resilience, fractal dimension, fracture morphology

## Abstract

In order to obtain high-performance 3D printed parts, this study focuses on the key performance indicator of impact toughness. The parametric modeling software Rhino 6 is used to design impact specimens, and the laser selective melting equipment DiMetal-100, independently developed by the South China University of Technology, is used to manufacture impact specimens. Subsequently, the CoCrMo alloy parts were annealed using an MXQ1600-40 box-type atmosphere furnace and subjected to impact testing using a cantilever beam impact testing machine XJV-22. Fractal theory was applied to analyze the fractal behavior of the resulting impact fracture surfaces. The research results indicate that the 3D-printed impact specimens exhibited excellent surface quality, characterized by brightness, low roughness, and the absence of significant defects such as warping or deformation. In terms of annealing treatment, lower annealing temperatures did not improve the impact performance of SLM-formed CoCrMo alloy parts but instead led to a decrease in toughness. While increasing the annealing temperature can improve toughness to some extent, the effect is limited. Furthermore, the relationship between impact energy and heat treatment temperature exhibits a U-shaped trend. The fractal dimension analysis shows that the parts annealed in a 1200 °C furnace have the highest fractal dimension and better toughness performance. This study introduces a novel approach by comprehensively integrating advanced 3D printing technology, annealing processes, and fractal theory analysis to systematically investigate the influence of annealing temperature on the impact properties of 3D-printed CoCrMo alloy parts, thereby establishing a solid foundation for the application of high-performance 3D printed parts.

## 1. Introduction

Toughness, a concept in physics, refers to a material’s ability to absorb energy during plastic deformation and fracture. The better the toughness, the less likely a brittle fracture is to occur. In the fields of material science and metallurgy, toughness refers to a material’s ability to resist fracture when subjected to a force that deforms it. It is defined as the ratio of the energy that can be absorbed by a material prior to fracture to its volume [1]. When developing new high-performance medical devices, they are usually designed as biomimetic structures to improve performance. The influence of material toughness on biomimetic structures is mainly reflected in improving overall mechanical performance, suppressing crack propagation, and providing inspiration for optimized design. Materials with good toughness exhibit higher strength and plasticity. In biomimetic structures, the toughness of materials is crucial for improving the overall strength and toughness of the structure. Materials with good toughness can absorb more energy when subjected to external forces, thereby delaying the propagation of cracks. In biomimetic structures, this toughness effect is further enhanced. The specific spatial configuration with constantly changing micro orientations in biomimetic structures can induce cracks to deflect along the biomimetic structure, increasing the area of the crack surface. The toughness of materials is directly related to the durability, impact resistance, and service life of medical device products and has a direct impact on the treatment effect and safety of patients. In view of this, studying the toughness of 3D-printed parts is of great significance for their application in medical treatment and other fields.

3D printing technology, based on the concept of “discrete/stack forming”, involves a process where materials are “stacked” layer by layer to form solid parts. This process is also known as “rapid prototyping” or “additive manufacturing” [2,3]. Three-dimensional printing technology offers multiple advantages, including the manufacturing of complex structural parts, the increase in productivity, the reduction of production cost, higher precision, the diversity of materials, the optimization of material properties, and a wide range of applications. Selective laser melting (SLM) molding is a 3D printing technology that melts metal powders using laser energy [4,5].

Zhong Zhi et al. [6] optimized the structure of conventional SLM additive manufacturing devices to meet the additive manufacturing needs of magnesium alloy materials. Taking AZ91D magnesium alloy spherical powder dedicated for additive manufacturing as the raw material, they additively manufactured AZ91D magnesium alloy powder using an improved SLM device. To explore the effect of galvanometer precision on SLM-formed parts, Liu Pengyu et al. [7] prepared Ti6Al4V samples at three different galvanometer precisions and analyzed the hardness, density, phase, microstructure, mechanical properties, and fracture morphology of the formed parts. The results suggested that the phases of all three groups of samples were α/α’. With the deterioration of galvanometer precision, the density, Vickers hardness, yield strength, and strain value of the samples showed a downward trend. Yan Chao et al. [8] fabricated pure tantalum samples using SLM technology, analyzed the surface quality and density of tantalum samples fabricated under different process parameters, determined the optimal parameters, and analyzed the microstructure and mechanical properties of the samples with optimal parameters. The results suggested an average tensile strength of 672 MPa, a yield strength of 623 MPa, and an elongation at a break of 23.2%. Irregular parts fabricated under these process parameters showed excellent quality when annealed at temperatures between 950–1450 °C and held for 120 min, without cracks or defects in the finished products. These parameters represented the optimal parameter combination. Using an electronic universal material tester, Xiang Chao et al. [9] carried out a tensile test on 18Ni300 maraging steel samples undergoing different heat treatments. Following heat treatment, the melt pools of samples gradually disappeared, and the characteristics of the martensitic structure became more pronounced. The hardness grew from 34.1 HRC to 52~54 HRC, while the tensile strength grew from 1174 MPa to more than 2000 MPa. Taking the GH4169 high-temperature alloy prepared by SLM technology as the research object, Zhao Haisheng et al. [10] designed two different heat treatment regimes. The results indicated that homogenization heat treatment eradicated the Laves phase, resulting in a transverse-to-longitudinal high-temperature tensile strength ratio and a ductility ratio of 1.00 (1041/1038) and 1.00 (8.6/8.6), respectively. This process effectively eliminated the anisotropy in both the microstructure and mechanical properties of the SLM-formed GH4169 alloy. Wang Yangyang et al. [11] investigated the effect of heat treatment temperature on the impact toughness and anisotropy of 316L stainless steel. They used selective laser melting (SLM) technology to prepare 316L impact components with different forming orientations and compared the impact toughness and microstructure of the SLM state, 1050 °C heat treatment state, and 1100 °C heat treatment state. The results show that, arranged in descending order of impact toughness, as the heat treatment temperature increases, the toughness dimples on the impact fracture surface gradually increase. After heat treatment, the subgrain structure gradually disappears, inclusions coarsen, grain size increases, and large-angle grain boundaries increase. The multi-layer structure prepared layer by layer is destroyed, and the grain texture changes. These microstructural changes are closely related to impact toughness. Li Yanli et al. [12] analyzed the impact toughness of 304L stainless steel formed by laser selective melting under the conditions of laser power of 300–340 W and laser scanning speed of 800–1500 mm·s^−1^. They found that under the condition of laser energy density of 100–140 J/mm^3^, the impact toughness of 304L remained stable at around 138 J. Zhang Guoqing et al. [13] found that SLM-formed CoCrMo alloy had higher elongation and tensile strength than national standards and castings of the same batch, following water cooling after a 2-h soak at 1200 °C. Ghayoor M et al. [14] explored the effect of volume energy density on the microstructure evolution, texture, and mechanical properties of 304L stainless steel parts fabricated through SLM. By utilizing SLM technology, Tolosa I et al. [15] investigated the mechanical properties of additively manufactured metal alloys and their variations with manufacturing direction (anisotropy). H. K. Rafi et al. [16] compared the microstructures and mechanical properties of Ti6Al4V parts prepared by SLM and electron beam melting (EBM), finding that SLM- and EBM-processed Ti6Al4V were different in microstructure evolution and mechanical properties. Bogahawattha et al. [17] designed a Menger fractal cube (MFC) and manufactured it using 3D printing technology. Performance tests showed that the third-order MFC exhibited the highest specific energy absorption, while the fourth-order MFC had the highest densification displacement and energy absorption efficiency. Macek et al. [18] studied the fractal dimension of the fatigue fracture surface of 18Ni300 martensitic aged steel manufactured by selective laser melting. They concluded that the geometric shape Df of the fracture plane represented by the fractal dimension can facilitate the estimation of the loading history after failure. Macek W further analyzed the fracture surface morphology of two types of steel and aluminum alloys under bending torsion fatigue loading and found a correlation between fractal dimension, other selected parameters of surface morphology (such as area Sx), and fatigue loading conditions [19]. We used fractal interpolation theory to analyze the influence of relevant parameters on the compression performance of the CoCrMo porous structure formed by laser-selective melting in the early stage. Our findings revealed that the elastic modulus decreases with the increase in porosity, the increase in average pore size, and the decrease in surface-area-to-volume ratio. Conversely, compressive strength increased with decreasing porosity and average pore size and increasing surface-area-to-volume ratio [20].

While significant research has explored the performance of 3D-printed metal parts, partially validating their reliability, further improvements are needed, especially in terms of impact toughness after heat treatment. The influence mechanism of different heat treatment processes on the microstructure and mechanical properties of 3D printed metal parts is not fully clear. How to improve the impact toughness of 3D printed metal parts by optimizing heat treatment processes has become a pressing issue. This article, therefore, mainly focuses on the impact toughness of 3D-printed metal parts after heat treatment.

## 2. Materials and Methods

### 2.1. Design Methods

**Figure 1:**
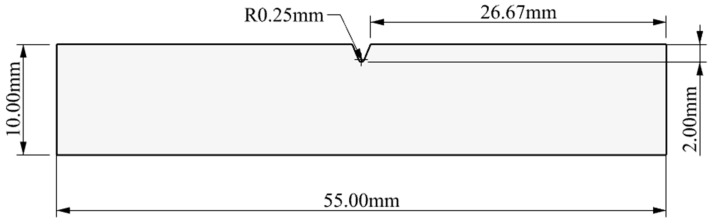
V-notch specimen.

According to the dimensions specified in the GB/T229 standard, an impact sample was designed, and related test properties were analyzed based on this standard [21]. For this purpose, we completed the design of the impact sample in Rhino 6 software developed by Robert McNeel & Associates in the United States, with overall dimensions of 10 mm × 10 mm × 55 mm. Figure 1 shows the designed impact sample as well as its dimensions. The impact sample was required to have a machining tolerance of ±0.11 mm and a surface roughness of less than 5 μm.

### 2.2. Materials and Manufacturing Methods

**Figure 2:**
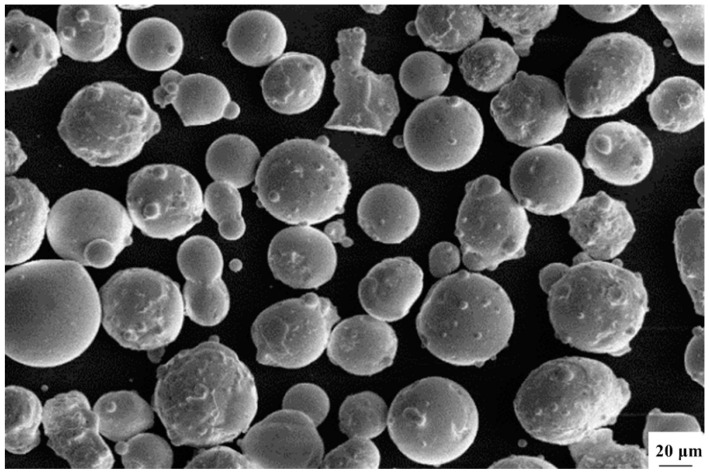
Microscopic morphology of CoCrMo alloy powder.

The material intended for SLM-formed impact samples was cobalt-chromium-molybdenum (CoCrMo) alloy powder, produced by SANDVIK Osprey in the UK, with powder composition meeting the ASTM F1377 standard. A comparison of the composition test results is presented in Table 1. The powder produced by aerosolization exhibited a spherical shape, as shown in Figure 2. Using the JL-1177 fully automatic laser particle size analyzer from Chengdu Jingxin Powder Testing Equipment Co., Ltd. in Chengdu, China, it was found that the powder particle size exhibited a narrow and concentrated distribution. 90% of the powder particles with a size less than 22.24 μm could pass through the sieve, and the average particle size of D50 powder was 28.5 μm.

The impact sample was then fabricated using a DiMetal-100 SLM device developed by the South China University of Technology. With nitrogen as the protective gas, the oxygen content was controlled below 0.03%, with a laser processing power of 170 W, a scanning speed of 500 mm/s, a scanning spacing of 60 µm, and a layer thickness of 35 µm, utilizing an X-Y interlayer interlaced scanning strategy.

### 2.3. Analysis Methods

The box type atmosphere furnace MXQ1600-40 produced by Dongguan Zhiyuan High Heat Machinery Technology Co., Ltd. in Dongguan, China was used for annealing CoCrMo alloy parts, with a heating rate of 8 °C/min and nitrogen protection. After heating to the set temperature and holding for 2 h, furnace cooling or air cooling was performed, respectively. An XJV-22 cantilever beam impact tester was utilized for the impact test, with an impact speed of 3–6 m/s. The SEM equipment MERLIN produced by Carl Zeiss AG in Oberkochen, Baden-W ü rttemberg, Germany was used to capture fracture images of the formed parts and analyze their fracture mechanism.

Fractal theory uses the perspective and mathematical methods of fractional dimension to describe and study objective things, that is, using mathematical tools of fractal dimension to describe and study objective things, which has advantages in revealing the regularity of irregular and self-similar structures. The fractal theory has great applications in the fields of defect initiation, damage evolution, crack propagation, instability and fragmentation in metals, rocks, and other materials. Therefore, this article will apply fractal theory to analyze the fractal behavior of impact fracture surfaces.

**Figure 3:**
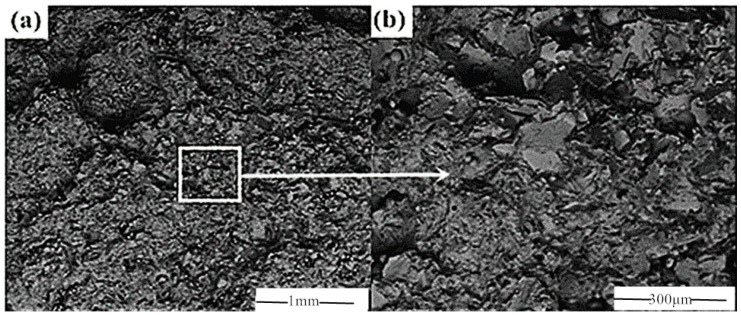
Tensile fracture image of CoCrMo alloy: (**a**) SEM at 80× magnification; (**b**) SEM image of image (**a**) magnified 300 times locally.

The SEM image of tensile fracture of CoCrMo alloy is depicted in Figure 3. From Figure 3, it can be seen that although there were differences in details between the locally enlarged SEM image and the original image, the overall morphology remained largely consistent, indicating a high degree of similarity.

The complexity and self-similarity of a fractal were measured via fractal dimension. There were 4 fractal dimensions in the Euclidean space (D = 0, 1, 2, 3). There were many methods for calculating fractal dimensions, including the box-counting method, fractional Brownian motion, and area measurement. The box-counting method, proposed by Russel et al. [22], is widely used due to its simplicity and convenience, etc. Therefore, in this paper, the box-counting method was mainly used to calculate the fractal dimensions of the SEM image of the impact fracture.

Let A be any non-empty bounded subset of R^n^ space. For any r > 0, N_r_(A) represented the minimum number of n-dimensional cubes (boxes) needed to cover the side length r. If there existed a number D such that when r→0:

(1) $$N_{r}(A)\infty 1/r^{D}$$

then D was called the box-counting dimension of A. Additionally, if and only if there existed a unique positive number k such that:

(2) $$\underset{r\to 0}{\lim}\frac{N_{r}(A)}{1/r^{D}}=k$$

Taking the logarithm of both sides of Equation (2), we can derive the following calculation:

(3) $$D=\frac{\ln N(r)}{\ln(1/r)}$$

where D was the self-similar dimension;

Nr was the number of boxes;

r was the length of the yardstick.

The relationship between fractal dimension and impact energy can be established using the least-squares fitting and regression analysis methods, which can directly infer the fracture properties of metals from the fractal dimension analysis of the fracture surface after metal failure. The specific process is as follows: (1) Import the fractal dimension and impact energy data calculated by the above method into Origin 2018 software; (2) Select Linear Fit for least-squares fitting in Origin software; (3) Perform data processing on the fitted curve equation to obtain the corresponding equation.

## 3. Results and Discussion

### 3.1. Data Processing for the 3D-Printed Impact Sample

**Figure 4:**
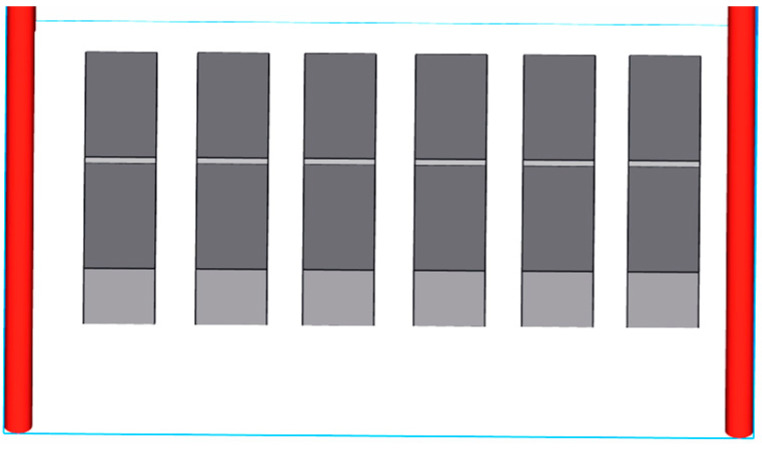
Data processing of 3D printed impact specimens.

The different placement and support addition methods of 3D-printed parts can directly affect the forming quality and manufacturing cost of the parts, so it was necessary to study them. For the impact sample, this paper adjusted the placement of the impact samples based on previous findings on the placements and support additions of SLM-formed parts [21]. Due to the excessive length of the impact samples, we utilized a placement strategy where the notch was laid flat, facing down, and tightly contacting the base plate to prevent warping and deformation, as shown in Figure 4.

### 3.2. Analysis of the Effectiveness of 3D-Printed Parts

**Figure 5:**
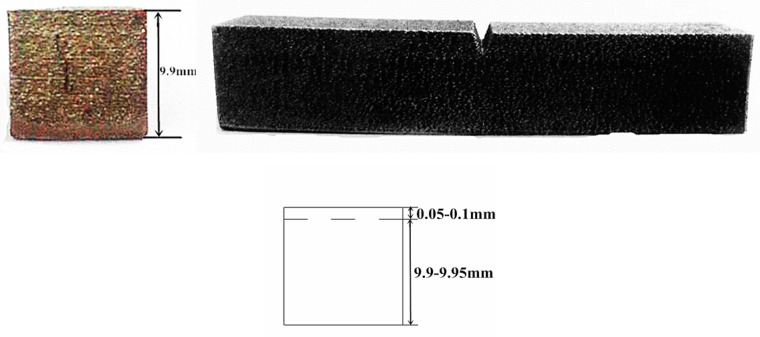

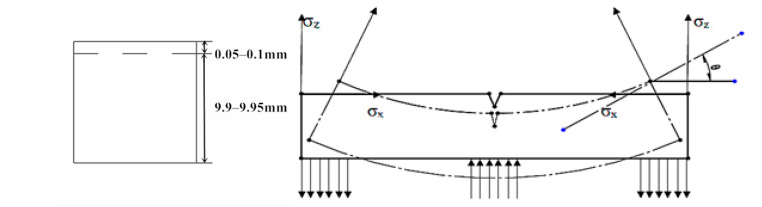
Impact test specimens completed by 3D printing.

The impact sample fabricated using 3D printing technology is displayed in Figure 5. By observing Figure 5, we can find that the 3D-printed impact sample presented a shiny surface with low roughness and no obvious defects, such as warping or deformation. The height of the impact sample was measured to be about 9.9 mm using a caliper perpendicular to the manufacturing direction. After removing the influence of powder adhering to the surface of the parts, the error in the height direction was about 0.05–0.1 mm, mainly owing to dimensional errors of the formed parts caused by the SLM forming mechanism or other factors. Since the dimensional errors for the same batch of 3D-printed parts were similar, this did not affect the comparative analysis of the sample’s impact resistance.

### 3.3. Analysis of the Impact Performance of 3D-Printed Parts

**Figure 6:**
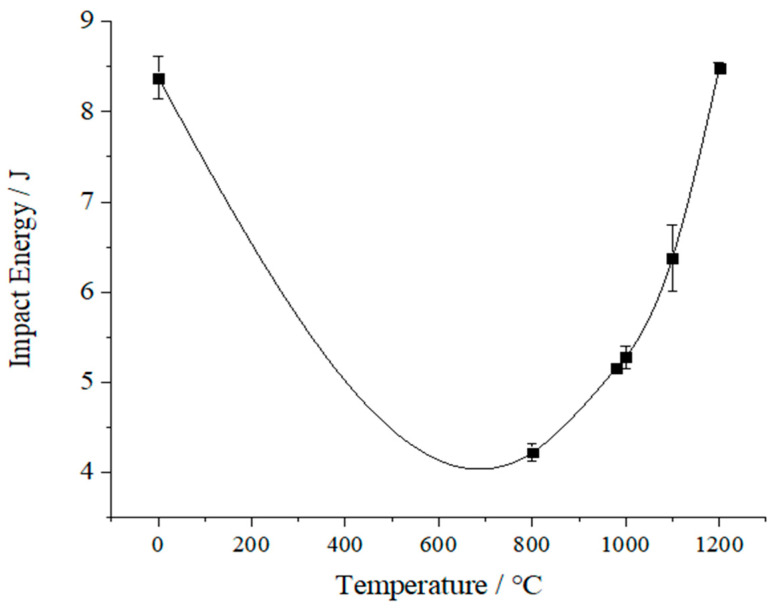
Curve of impact energy with temperature change.

An impact test was conducted according to the impact test method outlined in Section 2.3. The impact energy results of the SLM-formed CoCrMo alloy impact samples are shown in Table 2, with three samples in each group. From Table 2, it was evident that all SLM-formed parts exhibited certain impact toughness before annealing. The samples annealed at 980 °C and 1000 °C show the lowest impact energy values, with the 1100 °C annealed samples showing slightly greater impact energy than those annealed at 1000 °C. The highest impact energy was observed for the sample annealed at 1200 °C. This suggested that lower annealing temperatures cannot improve the impact performance of SLM-formed CoCrMo alloy parts but instead reduce their toughness. Increasing the annealing temperature did bring some improvement to the toughness of CoCrMo alloy parts, but the effect was not remarkable, possibly due to factors like holding time and cooling method. In order to analyze the relationship between impact energy and heat treatment temperature, a curve fitting was carried out on impact energy and heat treatment temperature, as shown in Figure 6. As manifested in Figure 6, there was a U-shaped relationship between impact energy and heat treatment temperature, suggesting the presence of a ductile-to-brittle transition temperature between heat treatment and impact energy. The impact toughness (ak) of the SLM-formed parts was calculated by dividing the impact energy (Ak) by the cross-sectional area (F) at the notch of the SLM-formed impact sample, i.e., ak = Ak/F, where ak is measured in J/cm^2^.

### 3.4. Analysis of the Fractal Behavior of Impact Fracture

Traditionally, there were two ways to select box sizes: geometric sequence and arithmetic sequence. In practical calculation, however, boundary effects often arose from improper box size selection. In this paper, the box size was chosen mainly based on factor sequence (greatest common divisor). For example, if the size of the cropped SEM image was 120 × 120, then the geometric sequence was Sgcd = {1, 2, 4, 8, 16 …}, and the arithmetic sequence was chosen with a constant step of 4, resulting in Sgcd = {1, 5, 9, 13, 17 …}. The factor sequence utilized in this study was Sgcd = {1, 2, 3, 4, 5, 6, 12 … 120}, which precisely divided the image integrally without any boundary effect.

The process of calculating the fractal dimension in MATLAB involves the following steps:

(1) In the first place, crop, filter, and threshold-segment the SEM-captured fracture image and convert it into a binary image.

(2) Cover the processed SEM fracture image with different box sizes (r) selected via factor sequence.

(3) Calculate the number of boxes Nr containing the image.

(4) Take the logarithm of the reciprocals of the box count Nr and box size (r) for different size coverages and carry out a least-squares fitting, where the slope of the fitted line was the fractal dimension.

**Figure 7:**
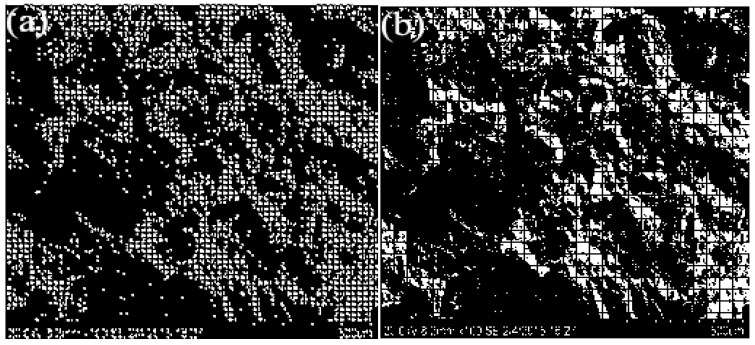
SEM images of impact fracture under different scale box covers: (**a**) SEM image under 1 mm box cover (**b**) SEM image under 1.5 mm box cover.

Figure 7 presents the results of the box-counting method in MATLAB 2018, where different box sizes (r) were used to cover the SEM images of impact fractures. By calculating the number of boxes under different size coverages and then performing curve fitting, the fractal dimension can be derived. Algorithm 1 shows the basic structure of the box-counting method code.


**Algorithm 1** Basic structure of box algorithm code.Start Read SEM image % input the image  level = graythresh(I);  BW = im2bw(I,level); % image binaryzation  I2 = imcrop(BW,[181 181 489 489]); % image clipping Step (2) for j = 1:x   for k = 1:y  fprintf(fid,‘%d’,I(j,k)); % image data preservation    end  fprintf(fid,‘\n\n’);    end Step (3) nn = length(im);  thresh = 120;  im = (im > thresh); % read the sizes of the image  [M,N] = size(im); Step (4) Ngcd = gcd(M,N);  Nmin = min([M,N]);  if Ngcd == Nmin % greatest common factor  Sgcd = Nmin;    else  Sgcd = Ngcd;  for a = 1:Sgcd  for(i = 1:k:M)  nr = nr + nj; % count the total number Step (5) xd = get(h,‘XData’);    yd = get(h,‘YData’); % least square fitting  a = [yd(1) − yd(2)]./[xd(1) − xd(2)] % fractal dimension    b = yd(1) − a*xd(1)


According to classical fracture mechanics and quantitative fracture morphology analysis, it was known that rougher surfaces required more energy consumption during the fracture process. Therefore, the rougher the surface of the fracture, the greater the fractal dimension, and a higher fractal dimension typically indicates better toughness of the part [23,24,25,26].

Considering different fractal dimensions at macro and micro levels, the toughness in macroscopic images increased with the fractal dimension, while the toughness in microscopic images decreased with the fractal dimension. This study took SEM macroscopic images magnified by a factor of 300 for comparative analysis.

**Figure 8:**
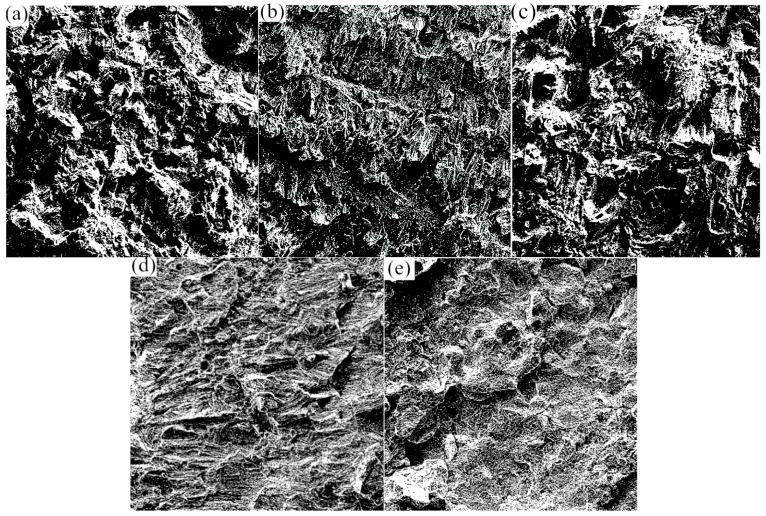
Binary SEM image: (**a**) Untreated fracture image; (**b**) 800 °C heat treatment fracture image; (**c**) 980 °C heat treatment fracture image; (**d**) 1100 °C heat treatment fracture image; (**e**) 1200 °C heat treatment fracture image.

Figure 8 illustrates the SEMs of the impact fractures after contrast adjustment, cropping, filtering, denoising, and binarization. In Figure 8, (a) shows the untreated fracture; (b) displays the fracture after heat treatment at 800 °C; (c) exhibits the fracture after heat treatment at 980 °C; (d) illustrates the fracture after heat treatment at 1100 °C; and (e) showcases the fracture after heat treatment at 1200 °C. In order to eliminate ineffective parts at the edges of the SEM images, the original image was cropped to a size of 489 × 489 for ease of MATLAB calculation. A binarization threshold of 120 was applied. The white part in Figure 8 was the raised part, and the black part was the depressed part. Combined with Table 2, as the impact toughness of the SLM-formed parts improved, the white part in the binarized SEMs of impact fractures increased, indicating a trend in fractal dimension changes where the toughness grew with larger fractal dimension.

**Figure 9:**
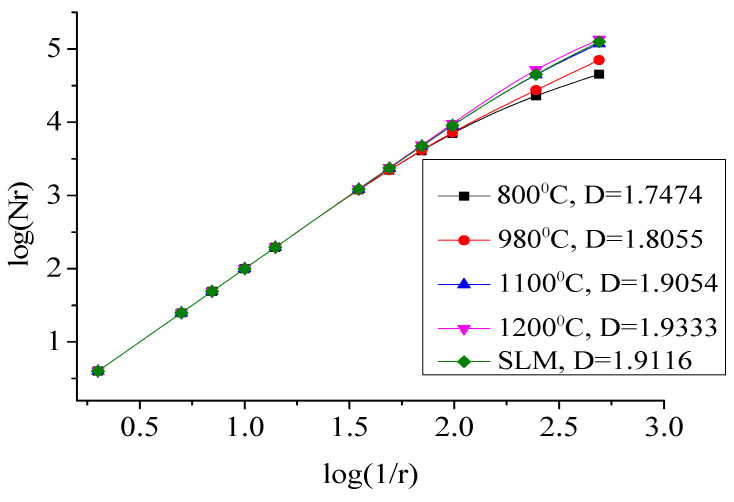
Fractal dimension curve of impact fracture surface.

Figure 9 displays the fractal dimension curve of impact fractures, with Nr representing the number of boxes and r representing the length of the yardstick. After taking the logarithm, a least-squares fitting was performed to determine the fractal dimension. The fractal dimension curve exhibited good linearity, with all correlation coefficients exceeding 98% post-least-squares fitting, highlighting the significant fractal behavior on the surface of impact fractures. The fractal dimension increased with rising annealing temperature. The fractal dimensions were 1.9116 for the untreated sample, 1.7474 after annealing at 800 °C, 1.8055 after annealing at 980 °C, 1.9054 after annealing at 1100 °C and furnace cooling, 1.9333 after annealing at 1200 °C and furnace cooling, and 1.9116 after annealing at 1200 °C and furnace cooling. The untreated sample showed a higher fractal dimension than samples annealed at 800 °C, 980 °C, and 1100 °C. The part annealed at 1200 °C and cooled in a furnace demonstrated the highest fractal dimension, indicating greater irregularities on its surface. Surface irregularities were often related to the presence of dimples and shear lips on the material surface, while the magnitude of the impact energy was also often related to the presence of dimples and shear lips on the material surface. Thus, a relationship can be established between impact energy and fractal dimension, allowing for the inference of impact energy from the fractal dimension.

**Figure 10:**
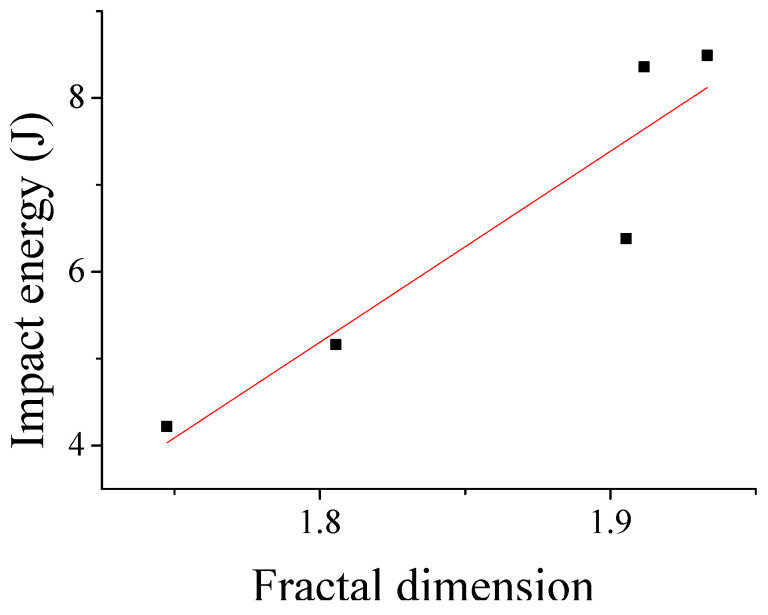
Relationship between fractal dimension and impact power.

The least-squares fitting of the fractal dimension and impact energy yielded a relationship curve between fractal dimension and impact energy, as shown in Figure 10. The correlation coefficient after fitting was 98.6%, indicating a fairly accurate reflection of the correlation between impact energy and fractal dimension and the correlation direction. Figure 10 demonstrated that the fractal dimension was proportional to the impact energy, and the fractal dimension increased with rising impact energy. The regression analysis of fractal dimension and impact energy curve yields Equation (4); therefore, the impact energy can be directly inferred from the fractal dimension of the fracture surface.

Y = 21.989X − 34.3921(4)

## 4. Conclusions

This study conducted a systematic and in-depth exploration of the impact performance of 3D printed CoCrMo alloy parts, leading to several innovative and practical findings. Firstly, regarding the surface quality of 3D printed specimens, this study confirms that 3D printing technology can prepare impact specimens with excellent surface quality, brightness, and low roughness without significant warping deformation. While dimensional inaccuracies (approximately 0.05–0.1 mm in the build direction) primarily stem from the inherent forming mechanism, this observation provides valuable insights for optimizing 3D printing process parameters and improving part size accuracy.

Furthermore, this study thoroughly analyzed the effect of annealing temperature on the impact performance of CoCrMo alloy parts formed by SLM. Research has found that annealing temperature has a significant impact on the impact toughness of parts: annealing at lower temperatures reduces the toughness of materials, while higher temperatures can improve toughness to some extent, but the effect is limited. More importantly, a U-shaped relationship exists between impact energy and heat treatment temperature, which may be closely related to insulation time and cooling method.

In addition, this study also revealed the intrinsic relationship between surface morphology and mechanical properties through fractal dimension analysis. The results showed that the as-printed sample exhibited the highest fractal dimension (1.9116), while after cold treatment in a 1200 °C annealing furnace, the fractal dimension increased to 1.9333, indicating that the surface roughness of the sample reached its maximum under this treatment condition. This finding provides a new quantitative indicator for evaluating the surface quality of 3D printed parts, but also a novel perspective for understanding the mechanism by which surface morphology affects mechanical properties.

Compared with existing literature, this study not only gained new insights into the impact performance of 3D printed CoCrMo alloy parts but also delved into the correlation between surface morphology and mechanical properties, employing fractal dimension analysis and other methods. These findings not only provide important guidance for optimizing 3D printing process parameters and improving part performance but also offer new ideas and avenues for future research. In the future, the continuous advancement of 3D printing necessitates in-depth research into the mechanisms by which process parameters influence surface quality and mechanical properties, alongside exploration of torsional performance in diverse parts and fracture toughness under mixed loading, all of which are critical for enabling the direct manufacturing of high-performance components through 3D printing.

## Figures and Tables

**Table 1:** The comparison of powder material manufactured in SLM and ASTM F75 standard.

Element	CoCrMo Powder	ASTM F75 Standard	Element	CoCrMo Powder	ASTM F75 Standard
Cr	29.4%	27–30%	C	0.15%	<0.35%
Mo	6%	5–7%	Ni	0.09%	<0.5%
Si	0.8%	<1%	Al	<0.010%	<0.1%
Mn	0.75%	<1%	Ti	<0.010%	<0.1%
Fe	0.26%	<0.75%	W	<0.010%	<0.2%
N	0.19%	<0.25%	Co	Balance	Balance

**Table 2:** Impact value list of specimens without heat treatment and after annealing treatment.

Sample	Impact Energy (J)			Impact Energy Average (J)	Impact Toughness (J/cm^2^)
Untreatments	8.13	8.6	8.4	8.36	11.91 × 10^−3^
Annealing (800 °C)	4.12	4.31	4.23	4.22	6.03 × 10^−3^
Annealing (980 °C)	5.14	5.16	5.17	5.16	7.83 × 10^−3^
Furnace cooling (1000 °C)	5.41	5.16	5.27	5.23	7.83 × 10^−3^
Furnace cooling (1100 °C)	6.01	6.39	6.74	6.38	9.11 × 10^−3^
Furnace cooling (1200 °C)	8.42	8.53	8.51	8.49	12.13 × 10^−3^

## Data Availability

The datasets generated during and/or analyzed during the current study are not publicly available due to confidentiality requirements but are available from the corresponding author upon reasonable request.
